# Mouse N2a Neuroblastoma Assay: Uncertainties and Comparison with Alternative Cell-Based Assays for Ciguatoxin Detection

**DOI:** 10.3390/md21110590

**Published:** 2023-11-13

**Authors:** Sandra Raposo-Garcia, Alejandro Cao, Celia Costas, M. Carmen Louzao, Natalia Vilariño, Carmen Vale, Luis M. Botana

**Affiliations:** Departamento de Farmacología, Facultad de Veterinaria, Universidade de Santiago de Compostela, 27002 Lugo, Spain; sandra.raposo.garcia@usc.es (S.R.-G.); alejandro.cao.cancelas@usc.es (A.C.); celia.costas.sanchez@usc.es (C.C.); mcarmen.louzao@usc.es (M.C.L.); natalia.vilarino@usc.es (N.V.)

**Keywords:** ciguatoxins, sodium channels, MTT, ciguatera fish poisoning, toxicity, LDH, Alamar blue

## Abstract

The growing concern about ciguatera fish poisoning (CF) due to the expansion of the microorganisms producing ciguatoxins (CTXs) increased the need to develop a reliable and fast method for ciguatoxin detection to guarantee food safety. Cytotoxicity assay on the N2a cells sensitized with ouabain (O) and veratridine (V) is routinely used in ciguatoxin detection; however, this method has not been standardized yet. This study demonstrated the low availability of sodium channels in the N2a cells, the great O/V damage to the cells and the cell detachment when the cell viability is evaluated by the classical cytotoxicity assay and confirmed the absence of toxic effects caused by CTXs alone when using the methods that do not require medium removal such as lactate dehydrogenase (LDH) and Alamar blue assays. Different cell lines were evaluated as alternatives, such as human neuroblastoma, which was not suitable for the CTX detection due to the greater sensitivity to O/V and low availability of sodium channels. However, the HEK293 Nav cell line expressing the α1.6 subunit of sodium channels was sensitive to the ciguatoxin without the sensitization with O/V due to its expression of sodium channels. In the case of sensitizing the cells with O/V, it was possible to detect the presence of the ciguatoxin by the classical cytotoxicity MTT method at concentrations as low as 0.0001 nM CTX3C, providing an alternative cell line for the detection of compounds that act on the sodium channels.

## 1. Introduction

Ciguatera fish poisoning (CF) is the most commonly reported foodborne illness related to the consumption of fish containing marine algal neurotoxins known as ciguatoxins. These toxins act by modulating voltage-gated sodium channels (VGSCs), specifically, they alter their functionality by acting on site 5 of their alpha subunit, making the channels open at resting cell membrane potentials [1]. CTXs are characterized by causing a negative shift in the activation voltage of sodium channels in a concentration-dependent manner reported in different in vitro models [2,3,4].

The most common symptoms of CF are gastrointestinal, such as nausea, vomiting, diarrhoea and abdominal pain. Cardiovascular disturbances, mainly bradycardia and hypotension, are reported after CTX consumption. Neurological symptoms such as paraesthesia of the lips and extremities, cold allodynia, ataxia and painful dysesthesias are also frequent [5]. Neurological sequelae derived from the consumption of fish containing CTXs may last from weeks to up to years. All CTX analogues play a critical role in the CF syndrome since, under some conditions, external sensory stimuli might trigger a ciguatera crisis due to the negative shift in the activation voltage of sodium channels [4]. It is often difficult to relate CF intoxication to the consumption of fish contaminated with ciguatoxins due to the wide variety and poor specificity of the ciguatera symptoms. The real number of ciguatera poisoning cases can reach up to 50,000–500,000 per year [6], but underreporting and misdiagnosis of the real CF cases were not contemplated in any study. There is no specific effective treatment either for acute or for chronic ciguatera poisoning. Supportive medical management of CF clinical effects is the available tool to fight against the CF manifestations [7].

CTX compounds are produced by benthic dinoflagellate species from the *Gambierdiscus* and *Fukuyoa* genera and enter the marine food chain through herbivorous fish and invertebrates [7]. CTXs were classified according to their geographical origin as Pacific ciguatoxins (P-CTX) identified in the Pacific Ocean, Caribbean ciguatoxins (C-CTX) first isolated in the Caribbean Sea and Indian ciguatoxins (I-CTX) found in the Indian Ocean. So far, P-CTXs have been the most studied compounds as a consequence of their abundance and distribution and were recently divided into two separate groups based on their chemical structure, the CTX3C group and the CTX4A group [7]. Among CTXs, P-CTX1 was considered the most potent analogue, and the recommended safety limit for CTXs in fish for human consumption has been set at 0.01 ng P-CTX-1 toxin equivalent/g of fish tissue by the United States Food and Drug Administration [8], a fact that has been recently refuted by the observation that the Caribbean analogue had similar potency [9], raising the need for the development of fast and sensitive methods to detect low levels of these compounds in food.

To monitor the levels of CTXs in fish flesh prior to sale and to decrease the probability of CTX consumption, many methods have been developed [10,11,12], but the complexity and variability of such toxins have made the development of a reliable method to routinely monitor CF with high specificity and sensitivity a real challenge. The current methodology available for CTX detection is very wide and diverse, from in vivo tests, not used due to the low availability of the compounds and ethical concerns, to cell-based assays, receptor-binding assays, liquid chromatography coupled with mass spectrometry (LC/MS) or immunoassays [13,14,15,16,17]. Currently, the most widely used methods to quantify CTXs are the cell-based assays because they are cheap, fast and easy to develop. Formerly, mouse bioassay (MBA) was the most commonly used method to evaluate the relative potency of CTXs as well as to detect their presence in food [18,19,20] despite its multiple disadvantages and ethical concerns [6]. However, the use of the MBA as a method to detect CTXs and therefore to evaluate their potency has the drawback such as that the data obtained are derived from establishing dose/survival-time relationships, and the end point of the assay was the death of the tested animal. Thus, death was considered as an indicator of CTX toxicity, while the main effect of CF is not the death but the symptoms [7,21]. 

Among the in vitro methods to detect CTXs in food, cell viability is commonly measured in the Neuro2a cell line from mouse by using the MTT (1-(4,5-dimethylthiazol-2-yl)-3,5-diphenylformazan) assay [22]. However, these marine toxins have no cytotoxic effect in non-excitable cells, such as undifferentiated neuroblastoma cells, and their detection requires the addition of the sodium/potassium (Na^+^/K^+^) ATPase pump blocker ouabain (O), together with the sodium channel activator veratridine (V) increasing the intracellular sodium load. Under this O/V treatment, the cytotoxic effect of CTXs was potentiated allowing for the detection of the presence of these toxins in samples [22]. However, despite being used at low concentrations, ouabain and veratridine have their own toxic effect on cells [12,23]. The N2a cell bioassay has not been validated at an international level yet because there is a high degree of variability in the literature regarding the conditions including the number of cells, amount of O/V, medium components and cell density [12]. Therefore, a large number of variations of this method have been developed over the years trying to improve its sensitivity and specificity [10,24]. 

Other detection methods for agents acting on the sodium channels have been developed and include the fluorometric detection of membrane depolarization as the indicator of the CTX presence [25] or the use of neuroblastoma cell lines such as SH-SY5Y [23] considered more appropriate due to their human origin. Moreover, the O/V treatment was demonstrated to cause damage in cells at concentrations from 100 to 10 μM O/V [12], but some studies were developed using O/V concentrations as high as 140/14 μM O/V [26], 220/22 μM O/V [24], 250/25 μM O/V [27,28,29] or even 500/50 μM O/V [30] or 1000/50 µM O/V [22]. Therefore, there is an increasing need to reevaluate the current methodology to detect ciguatoxins since they are emerging toxins in Europe whose detection at low levels in food is the key to protect the health of consumers. Thus, the present paper aims to compare different methods and different cell lines to improve the detection of CTXs, trying to clarify which method is more effective for this purpose.

## 2. Results

A wide range of experiments were developed to reevaluate the current cell-based assays available to detect the presence of ciguatoxins in food using three different cell lines. The experiments involved the routinely used mouse neuroblastoma N2a, the human neuroblastoma cell line SH-SY5Y that was purposed to be more suitable to mimic the toxicity in humans caused by CTXs [23] and the human HEK293 Na_v_1.6 cell line due to its overexpression of the human sodium channels. With the aim to determine the most suitable method to assess the toxicity of ciguatoxins, electrophysiological recordings and different methods to determine cell viability were studied. 

### 2.1. Action of CTXs in the N2a Cell Line 

Although the use of the mouse neuroblastoma N2a cell line to detect the presence of ciguatoxins has been routinely employed over the years [10,11,24,26,29,31,32], there is a lack of consensus on the development of a method for detecting agents acting on the sodium channels.

#### 2.1.1. Electrophysiological Recordings of Sodium Currents in N2a Cells 

**Figure 1:**
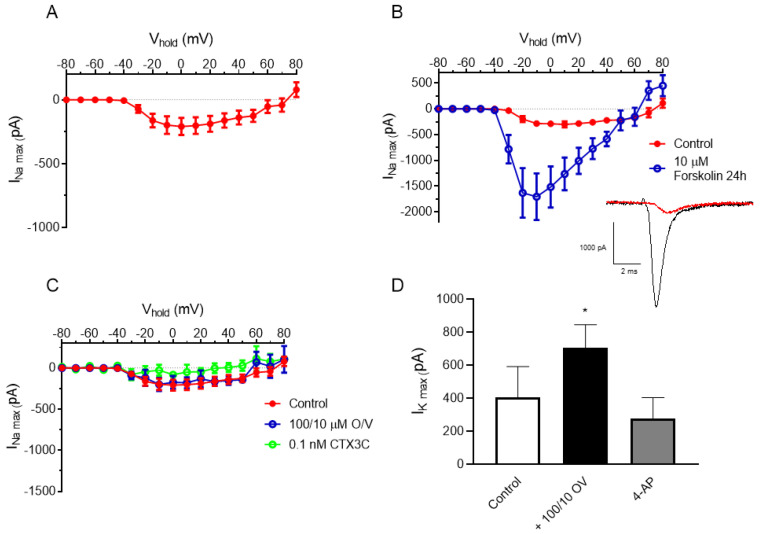
Sodium and potassium currents in N2a cells: (**A**) current–voltage relationship of sodium current amplitude in the N2a cell line without treatment with forskolin; (**B**) current–voltage relationship of sodium currents in N2a cells treated with 10 μM forskolin; (**C**) neither 100/10 µM O/V nor 0.1 nM CTX3C modified the current–voltage relationship for sodium currents in N2a cells (non-forskolin-treated); (**D**) increase in potassium currents after the addition of 100/10 µM O/V corroborated by the blockade with 15 mM 4-aminopyridine. Data are expressed as mean ± SEM (* *p* ≤ 0.05).

The sodium channel activity in the N2a cells was evaluated, and the results obtained were in agreement with previous studies showing low expression and availability of sodium channels in the N2a cell line [33,34,35]. The recording of the maximum sodium currents (I_Na_) in the undifferentiated N2a cells hardly reached −300 pA as shown in Figure 1A, demonstrating low sodium channel activity. In view of these results, the cells were exposed for 24 h to 10 μM forskolin, a molecule that elevate the intracellular concentration of the second messenger, cyclic adenosine monophosphate (cAMP), which plays a crucial role in cell differentiation [36,37,38,39,40,41,42]. Moreover, the activation of PKA by forskolin is known to increase the intracellular trafficking of several membrane-bound proteins (glucose transporter GLUT4, chloride channel CFTR, K_v_1.1 K^+^ channel and Na_v_1.5 Na^+^ channel) [43,44,45,46]. As indicated in Figure 1B, the current–voltage curve (I-V) showed an increase in the sodium current amplitude at all the recording potentials in the N2a cells treated for 24 h with forskolin. Thus, the maximum peak sodium current at −10 mV was −285 pA ± 50 pA in the control cells (*n* = 5) and −1706 ± 453 pA (*n* = 5) in the N2a cells pretreated with 10 μM forskolin for 24 h, demonstrating the low natural expression of sodium channels in the undifferentiated N2a cells. 

Since the N2a cell exposure to O/V is justified as a way to increase the sensitivity and specificity of the assay to compounds acting on VGSCs [24,32], electrophysiological recordings in the N2a cells were performed after the 24 h exposure of the cells to 100/10 μM O/V. As shown in Figure 1C, the cell exposure to 100/10 μM O/V for 24 h did not increase the intensity of the sodium channel currents, and further addition of 0.1 nM CTX3C to the recording chamber did not modify the sodium channel currents, demonstrating that the O/V cell treatment did not allow for the detection of CTX3C due to its direct effect on the sodium channels. Additionally, after the 24 h cell exposure to 100/10 µM O/V, an output potassium current was registered. The diminution of the current evoked after the addition of the known potassium channel blocker 4-aminopyridine at 15 mM, as shown in Figure 1D, confirmed the increase in the potassium current. This effect increasing outward potassium currents was, as expected, due to the effect elicited in the Na^+^/K pump by ouabain [47].

#### 2.1.2. Evaluation of the Effect of CTX3C Using the MTT Assay on the N2a Cell Line

**Figure 2:**
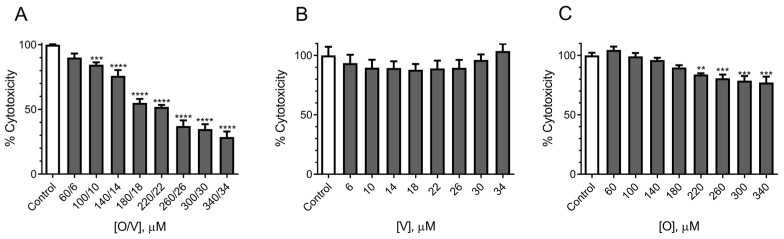
Cell viability of N2a cells assessed by the MTT test after 24 h exposure to the different combinations of O/V, veratridine alone and ouabain alone: (**A**) different O/V concentrations, (**B**) veratridine from 6 to 34 µM and (**C**) ouabain from 60 to 340 µM. Data are expressed as mean ± SEM of three independent replicates and as percentage of control cells (*n* = 3 in all the assays). Statistical differences were determined by ANOVA followed by Dunnet’s test (** *p* < 0.01, *** *p* < 0.001 and **** *p* < 0.0001).

The N2a cell viability was tested in the presence of O/V at concentrations ranging from 60/6 to 340/34 µM, since these proportions are usually employed in the literature for the screening of ciguatoxins. As shown in Figure 2A, the 24 h cell exposure to O/V elicited a concentration-dependent decrease in cell viability. These data indicated a cytotoxic effect of O/V in the cells, even at concentrations suggested not to have destructive effects in the N2a cells [12] since the combination of O/V at 100/10 µM elicited a decrease in cell viability from 100 ± 0.7% in the control conditions to 84.6 ± 1.8% (*n* = 3; *p* < 0.01) in the presence of O/V. To discriminate the independent cytotoxic effect produced in the cells by ouabain and veratridine, their cytotoxicity was evaluated separately. As illustrated in Figure 2B, veratridine did not cause alterations in the N2a cell viability after the 24 h exposure to concentrations from 6 to 34 µM. However, as shown in Figure 2C, the 24 h exposure of the N2a cell to ouabain concentrations of 220 µM and higher elicited a significant reduction in cell viability of 16.06 ± 2.41% (*n* = 3; *p* < 0.01) compared to the control conditions.

With these data, the IC_50_ values were calculated for the O and O/V conditions by fitting the data with a log (inhibitor) vs. normalized response model of cell viability (Supplementary Figure S1). The IC_50_ obtained for ouabain alone was 591.9 μM (confidence interval (CI): 430.2 to 814.5 μM) while the IC_50_ obtained for ouabain when combined with veratridine was 216.8 μM (CI: 205 to 229.2 μM) as shown in Supplementary Figure S1B based on the ouabain concentrations. 

**Figure 3:**
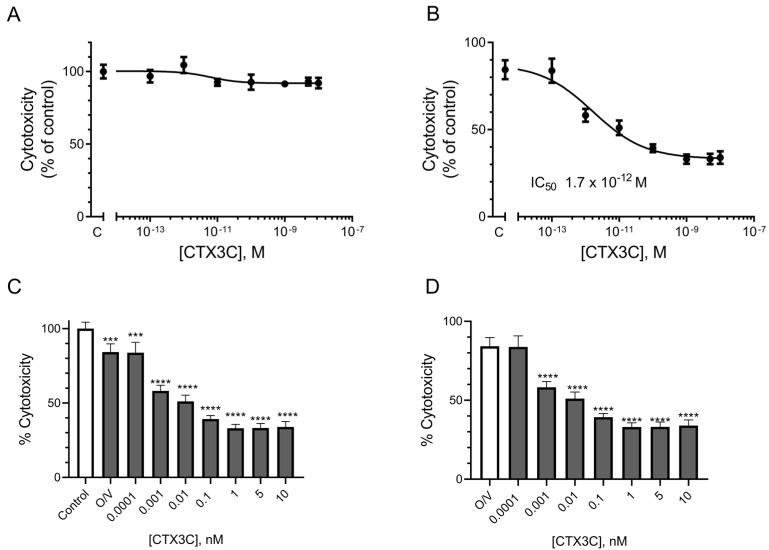
Cytotoxic effect of CTX3C assessed by the MTT method: (**A**) effect of CTX3C in N2a cells not exposed to O/V; (**B**) dose–response curve of N2a cells exposed to increasing concentrations of CTX3C in the presence of 100/10 μM O/V; (**C**) decrease in N2a cell viability after the exposure to 100/10 μM O/V and increasing CTX3C concentrations compared to control conditions; (**D**) decrease in N2a cell viability after the exposure to 100/10 μM O/V and increasing CTX3C concentrations compared to single 100/10 μM O/V treatment. (*** *p* < 0.001 and **** *p* < 0.0001).

The cytotoxicity caused by ciguatoxins in the N2a cells was studied in the absence and the presence of O/V. As shown in Figure 3A, no cytotoxic effect was observed after the cell exposure only to CTX3C in the absence of the O/V treatment. As expected, a concentration-dependent cytotoxic effect was detected after the cell exposure to 100/10 μM O/V with increasing CTX3C concentrations, yielding an IC_50_ value of 1.7 × 10^−12^ M (95% confidence interval (CI) from 4.7 × 10^−13^ to 5.837 × 10^−12^) by fitting the data with a log (inhibitor) vs. normalized response model, shown in Figure 3B. Figure 3C summarizes the cytotoxic effect of the 100/10 μM O/V treatment and further exposure to CTX3C (IC_50_ 6 × 10^−13^ M, 95% CI from 1.5 × 10^−13^ to 2.39 × 10^−12^ M); for this reason, the decrease in the cell viability due to the ciguatoxin was calculated as a function of the effect of the 100/10 μM O/V control value in Figure 3D. These data illustrate that the results obtained by performing the traditional MTT test on the sensitized N2a cells at our disposal were similar to those of different laboratories [9,32,48,49]. 

#### 2.1.3. Evaluation of the Effect of CTX3C Using the LDH Test in the N2a Cell Line

**Figure 4:**
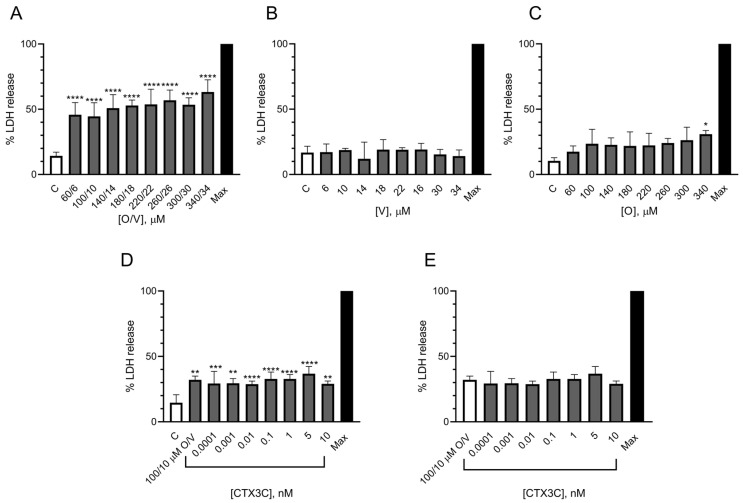
Cytotoxic effects of different O/V and CTX3C conditions after 24 h treatment in mouse neuroblastoma cells determined by the LDH assay: (**A**) cytotoxic effects of different O/V concentrations; (**B**) absence of cytotoxic effects of different V concentrations; (**C**) cytotoxic effects of different O concentrations; (**D**) cytotoxic effects of 100/10 μM O/V and different CTX3C concentrations compared to control conditions; (**E**) absence of cytotoxic effects of 100/10 μM O/V and different CTX3C concentrations compared to 100/10 μM O/V-treated cells. Data are mean ± SEM of four independent replicates. Statistical differences were determined by one-way ANOVA and with Dunnett’s multiple comparison test (* *p* ≤ 0.05, ** *p* ≤ 0.01, *** *p* ≤ 0.001, **** *p* < 0.0001). Max represents the maximum LDH release obtained following the manufacture’s instruction kit.

The LDH assay was performed to directly evaluate cell death due to the cell membrane damage elicited by different O/V concentrations. As shown in Figure 4A, even the lowest O/V concentration of 60/6 μM increased the LDH liberation by about 30% after the 24 h treatment. With the aim to further explore which compound is responsible for the cell damage, the cells were exposed to V or O separately, and the results are illustrated in Figure 4B,C, respectively. The results obtained show that whilst veratridine did not cause any increase in the LDH release, ouabain at the highest concentrations slightly increased the LDH liberation. Once the impact of the O/V cell treatment in the LDH release was known, the cytotoxic effect of CTX3C was evaluated in the presence of a commonly used concentration of 100/10 μM O/V which is considered nondestructive [12]. As shown in Figure 4D, when the percentage of the LDH release was compared with the spontaneous release in the control cells, all toxin concentrations increased the LDH liberation in the cells treated with 100/10 µM O/V. However, as illustrated in Figure 4E, when the LDH release was calculated as a function of the LDH release in the cells treated with 100/10 μM O/V and further exposed to CTX3C, there was no significant increase in the LDH liberation, evidencing that the cytotoxicity observed was due to the O/V treatment. No additional cell damage elicited by CTX3C was observed apart from that caused by the 100/10 μM O/V, indicating that this method does not allow for the detection of the presence of CTXs.

Due to the lack of consensus about a standardized protocol for the cellular assay on the N2a cell line, additional studies were performed by changing the cell culture conditions, specifically the FBS concentration and the number of seeded cells which were adjusted according to the LDH and Alamar blue protocols, to assess whether these parameters would affect the results obtained. As shown in Supplementary Figure S2, no differences were observed neither in the MTT assay nor in the LDH test with different cell culture conditions (seeded with a 5% FBS and treated with a 2% FBS supplemental medium). We concluded that the FBS concentration had no influence on the results obtained in none of the studies as described for the MTT assay [12]. For the cells treated with the 100/10 µM O/V and ciguatoxin for 24 h and cultured in a 10% FBS, an IC_50_ value of 1.7 × 10^−12^ M was obtained, very similar to the IC_50_ value of 6.3 × 10^−12^ M obtained for the cells under the same conditions but seeded with the 5% FBS and treated with the 2% FBS.

#### 2.1.4. Alamar Blue 

**Figure 5:**
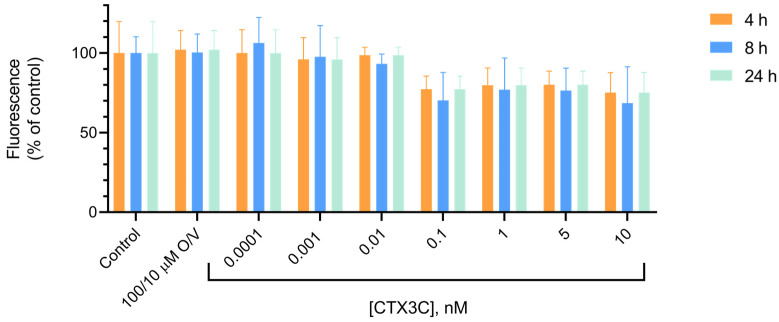
Alamar blue fluorescence at different times expressed as percentage of the control value, illustrating no significant decrease in fluorescence emitted by treated cells compared to control conditions. Statistical differences were determined by one-way ANOVA and with Dunnett’s multiple comparison test. All the determinations are the mean ± SEM of 3 independent culture plates in triplicate wells.

The Alamar blue method is widely used to determine cell viability after exposure to chemicals by a direct decrease in the fluorescence measurements over time in the culture plate with no need for medium removal. In this assay, the cells were exposed to 100/10 μM O/V and increasing CTX3C concentrations, and their viability was evaluated. Figure 5 illustrates the fluorescence measurements after the 4, 8 and 24 h cell treatment. The results obtained did not show any effect of CTX3C in the decrease in the cell viability after the 4, 8 or 24 h treatment.

#### 2.1.5. Direct Effects of CTX3C and Ouabain/Veratridine in the N2a Actin Cytoskeleton 

**Figure 6:**
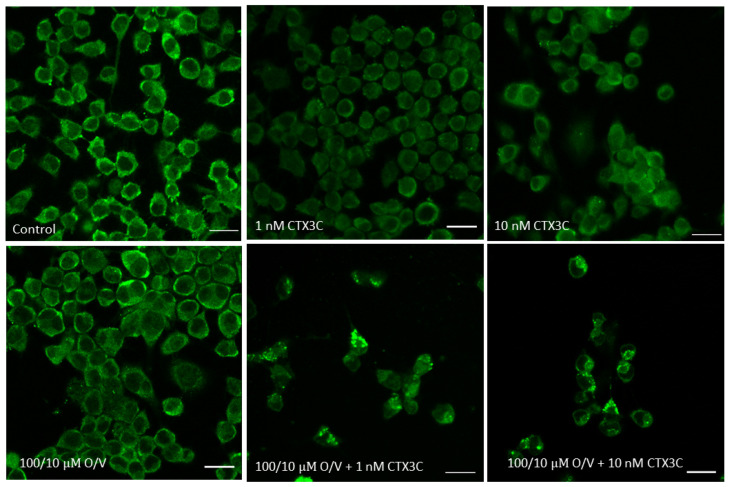
Representative confocal microscope images of the actin cytoskeleton stained in green in control N2a cells and in the same cells treated with either 1 or 10 nM CTX3C alone, 100/10 μM O/V or combinations of these with 1 or 10 nM CTX3C for 24 h in the culture medium. Scale bar is 20 μm.

Since in the initial experiments to evaluate the effects of O/V and CTXs on cells led to cellular damage and detachment, and previous works reported alterations in cytoskeleton caused either by CTXs [50,51] or by ouabain in different cell lines [52,53,54,55], the next approach to investigate the real effects of the O/V and CTX on the cells was by immunocytochemistry experiments marking the actin cytoskeleton. As shown in Figure 6, the 24 h exposure of the N2a cells only to 1 nM CTX3C caused changes in the cytoskeleton, giving a diffuse image of actin distribution, more pronounced at higher CTX3C concentrations (10 nM CTX3C), corroborating the damage to the cell cytoskeleton caused by CTXs alone. However, the actin cytoskeleton damage in the cells exposed only to 100/10 μM O/V was not noticeable, but when the cells were exposed for 24 h to 100/10 μM O/V and 1 or 10 nM CTX3C, the cells displayed spherical morphology and higher damage. It also highlights a remarkable decrease in cellular density as well as complete disruption of the actin cytoskeleton organization.

### 2.2. Increasing N2a Sensitivity to Ciguatoxins

**Figure 7:**
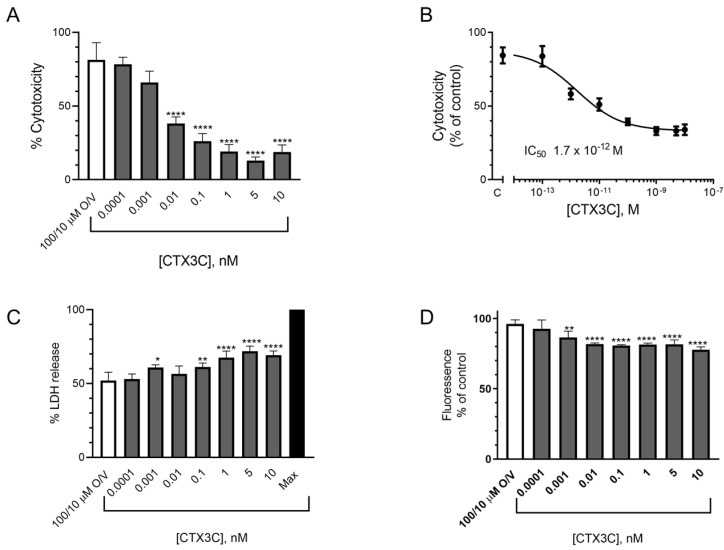
Effects of CTX3C on the viability of N2a cells pretreated for 24 h with 10 µM forskolin using different assays: (**A**) MTT assay; (**B**) concentration–response graph of the decrease in cell viability by increasing CTX3C concentrations as evaluated by the MTT assay; (**C**) cytotoxicity of CTX3C determined by the LDH assay; (**D**) cytotoxicity of CTX3C determined by the Alamar blue assay measured 24 h after Alamar addition. Statistical differences were determined by ANOVA followed by Dunnett’s multiple comparison test (* *p* < 0.05, ** *p* < 0.01 **** *p* < 0.0001, significantly different from the control).

In view of the remarkable increase in the sodium current amplitude of sodium channels obtained after the N2a cell exposure to 10 µM forskolin, further experiments were developed to evaluate if these effects correlated with increased sensitivity of the N2a cells to ciguatoxins. Once it was proved that the 24 h cell exposure to 10 µM forskolin was not toxic to the cells (Supplementary Figure S3), the cytotoxicity elicited by the ciguatoxin in the cells exposed for 24 h to 10 µM forskolin was evaluated by the MTT assay. As shown in Figure 7A,B, in the cells treated for 24 h with forskolin and thereafter exposed to 100/10 μM O/V and increasing CTX3C concentrations, a concentration-dependent decrease in the cell viability was obtained, leading to an IC_50_ of 4.3 × 10^−12^ M (CI from 2.4 × 10^−12^ to 7.9 × 10^−12^) by fitting the data with a log (inhibitor) vs. response (four parameters) model. Noteworthy, the cell viability decrease in this culture condition was higher than that in the non-forskolin-exposed cells, being 18.8 ± 2.4% at 10 nM CTX3C compared to 81.3 ± 5.8% observed in the presence of 100/10 μM O/V and compared to the decrease from 84.4 ± 5.5% in the control cells which were not pretreated to the 33.9 ± 3.6% viability in the presence of 10 nM CTX3C. 

When the cytotoxicity of the CTX in the cells pretreated with 10 µM forskolin was evaluated by the LDH and Alamar blue assays, the CTX cytotoxic effect was significantly increased compared to the non-forskolin-pretreated cells. As shown in Figure 7C, a significant increase in the LDH release was detected at doses of 0.001 nM CTX3C and above, reaching the levels of 69.2 ± 1.4% (*p* < 0.001) at 10 nM CTX3C compared to 52.0 ± 2.8% at 100/10 μM O/V. The results obtained with the Alamar blue assay illustrated in Figure 7D were very similar. In this case, the cells pretreated with 10 µM forskolin after the 24 h cell exposure to 100/10 µM O/V and increasing CTX3C concentrations showed a decrease in their metabolic rate. Thus, in the control conditions (single exposure to 100/10 µM O/V), the percentage of the cell viability was 96.2 ± 1.4% (*n* = 4) decreasing up to 86.5 ± 2.2% (*n* = 4) (*p* = 0.0025) when the cells were exposed to CTX3C at concentrations of 0.001 nM and higher. The highest CTX3C concentration evaluated, 10 nM, decreased the cell viability up to 77.6 ± 1% (*n* = 4). Therefore, the Alamar blue assay also allows for the detection of the presence of these compounds at low concentrations in the N2a cell pretreated with 10 µM forskolin for 24 h.

**Figure 8:**
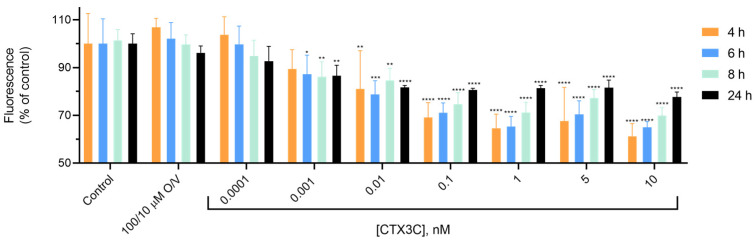
Alamar blue fluorescence at different times expressed as percentage of the fluorescence obtained in cells treated with 100/10 µM O/V after the addition of CTX3C at increasing concentrations. Statistical differences were determined by ANOVA test (* *p* < 0.05, ** *p* < 0.01, *** *p* < 0.001, **** *p* < 0.0001, significantly different from the control).

Since the Alamar blue assay allows for the measurement of fluorescence over time, the fluorescence measured at 4, 6, 8 and 24 h after the cell treatment (Figure 8) indicated that in forskolin-pretreated cells, the decrease in cell viability elicited by ciguatoxins could be detected at incubation times as brief as 4 h. A significant decrease in the cell viability was detected at 0.1 nM CTX3C with a decrease in fluorescence up to 69.1 ± 3.1% (*n* = 4) compared to 100 ± 6.3% (*n* = 4; *p* = 0.0008) in the control conditions and 106 ± 1.8% (*n* = 4) in the 100/10 μM O/V. After 6 h, 0.01 nM CTX3C was showed a decrease in fluorescence up to 78.7 ± 2.8% (*n* = 4; *p* = 0.0006), while at 8 h, CTX3C at 0.001 nM also showed a decrease in fluorescence up to 86.1 ± 3.1% (*n* = 4; *p* = 0.0009); decreases were expressed in all cases with respect to the fluorescence obtained in the O/V-treated cells.

### 2.3. Effect of CTX3C in the Human SH-SY5Y Neuroblastoma Cell Line

#### 2.3.1. Voltage-Gated Sodium Channel Functionality

Electrophysiological experiments in the SH-SY5Y cells were performed, and the results obtained highlighted that the undifferentiated neuroblastoma cells had very small sodium currents as previously reported [56]. In the undifferentiated SH-SY5Y cells, the maximum peak inward sodium current hardly reached −267 ± 0.29 pA at −10 mV (*n* = 3) as shown in Supplementary Figure S4 (the red line); however, the incubation of the SH-SY5Y cells with 10 μM forskolin for 24 h triggered an increase in the amplitude of sodium channel currents up to −1524 ± 246 pA (*n* = 6) as shown in Supplementary Figure S4 (the blue line).

#### 2.3.2. Evaluation of CT3C Cytotoxicity by MTT in Human Neuroblastoma Cells

**Figure 9:**
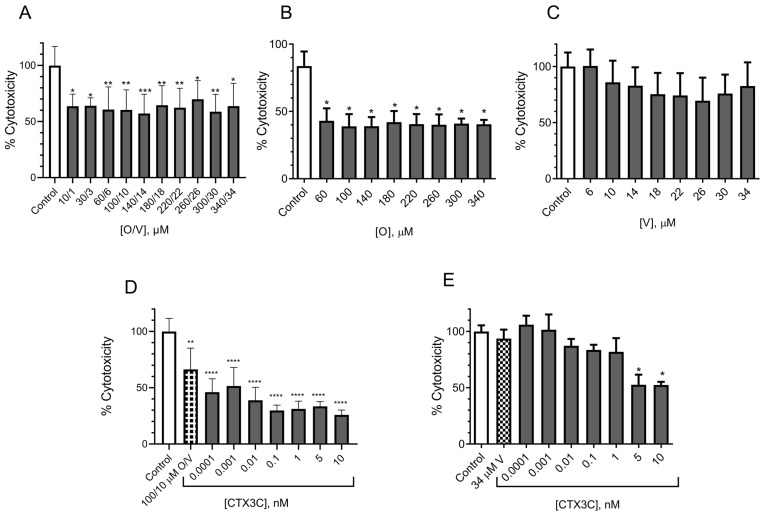
Cytotoxic effect measured by MTT assay on SH-SY5Y cells: (**A**) different O/V combinations; (**B**) different ouabain concentrations; (**C**) different veratridine concentrations; (**D**) O/V at 100/10 μM with different CTX3C concentrations; (**E**) veratridine at 34 μM with different CTX3C concentrations. Cell viability data are expressed as percentage of untreated control cells and the mean ± SEM of three independent experiments performed in triplicate (* *p* ≤ 0.05, ** *p* ≤ 0.01, *** *p* ≤ 0.001 and **** *p* ≤ 0.0001 versus control (untreated cells)).

Initially, the SH-SY5Y cell line was purposed as a potentially suitable cell model to evaluate CTX toxicity due to its human origin [23]. However, it has been previously reported that ouabain even at very low concentrations had a potent cytotoxic effect in this cell line [23]. With the aim to determine whether the human neuroblastoma cell line could be used with the MTT method for CTX detection, the toxicity of different O/V concentrations was analysed employing the same conditions as those described above for the N2a cell line. As shown in Figure 9A, the exposure of the SH-SY5Y cells to different O/V combinations decreased the cell viability by 36% at concentrations as low as 10/1 μM O/V and higher. When the O and V toxicity was assessed separately, as shown in Figure 9B, even the lowest O concentration employed, 60 μM, caused a decrease in the cell viability up to 60%. No significant decrease in the cell viability was observed when the cells were exposed to different V concentrations (Figure 9C). To mimic the same conditions as those in the previous experiments, the cells were coincubated at an O/V concentration of 100/10 μM. However, already at the proportion of 10/1 μM O/V, the cell viability decreased by 36%, a constant trend maintained after increasing the concentration ratios of O/V, which makes this method not suitable for the detection of ciguatoxins since considerable cell damage was elicited in the control cells, as summarized in Figure 9D. Since high V concentrations did not decrease the cell viability, 34 μM V was employed to enhance the CTX3C cytotoxicity with a compound that by itself does not cause the SH-SY5Y cell damage. The results obtained showed a significant decrease in the cell viability at CTX3C concentrations from 5 nM as shown in Figure 9E, indicating that this method does not allow for the detection of the presence of CTXs at lower concentrations.

The high sensitivity of the SH-SY5Y cell line to the O/V treatments was also confirmed by the LDH assay. As summarized in Supplementary Figure S5, O/V concentrations of 100/10 µM caused cytotoxic effects in the cells. The results obtained indicate that the human SH-SY5Y neuroblastoma cell line is not a suitable cell model for CTX detection due to its very low sodium channel expression, high ouabain sensibility and high CTX3C detection levels when the cells are incubated with veratridine. In view of these results and the previous findings reported by Coccini [23], no further experiments were developed with this cell line.

### 2.4. Evaluation of the Effect of CTX3C in HEK293 Cells Expressing the Human Na_v_1.6 Sodium Channel Alpha Subunit

The human HEK293 Na_v_1.6 cell line is an embryonic kidney cell line stably expressing the Na_v_1.6 subunit of sodium channels, which was used in previous reports to investigate the presence and potency of ciguatoxins as a function of their inhibition of the maximum peak inward sodium currents and the negative shift in their activation voltage [4,57,58]. Therefore, in the present work multiple studies were developed with this cell line to evaluate their accuracy as a new in vitro model to assess the toxicity of ciguatoxins.

**Figure 10:**
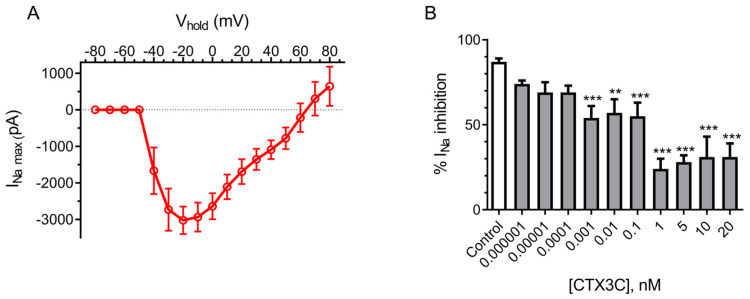
(**A**) Current–voltage relationship of sodium current amplitude in the HEK293 Na_v_1.6 cell line. (**B**) Inhibition of the maximum peak inward sodium currents by bath application of different CTX3C concentrations. Data are expressed as mean ± SEM and as percentage of inhibition of the sodium current (** *p* ≤ 0.01, *** *p* ≤ 0.001).

The electrophysiological recordings in HEK293 (Figure 10) showed a maximum peak inward sodium current of −2896 ± 476 pA (*n* = 8) which decreased in a concentration-dependent manner after bath application of increasing CTX3C concentrations (from 0.000001 to 20 nM), indicating a significant decrease in the maximum peak intensity at concentrations from 0.001 nM, as shown in Figure 10B, attributable to their hyperpolarizing effect on the activation voltage of sodium channels and the consequent intracellular increase in sodium [4]. However, electrophysiological recordings cannot be used routinely due to their requirement of special equipment and qualified staff to be developed. Nevertheless, electrophysiological techniques are the gold standard to detect both the changes that occur in ion channels; thus, these recordings are the proof to demonstrate that the HEK293 Na_v_ cell line transfected with the alpha subunit of voltage-gated sodium channels is suitable for the detection of ciguatoxins.

#### MTT in HEK Cells Expressing the Human Na_v_1.6 Sodium Channel Subunit

**Figure 11:**
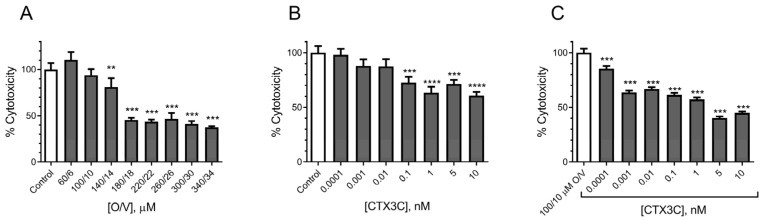
MTT assay on HEK Na_v_1.6 cells: (**A**) effect of selected O/V concentrations; (**B**) cytotoxic effect of increasing CTX3C concentrations alone; (**C**) potentiated cytotoxic effect of different CTX3C concentrations with 100/10 μM O/V. Cell viability data are expressed as percentage of untreated control cells. Data are the mean ± SEM of three independent experiments (** *p* ≤ 0.01, *** *p* ≤ 0.001 and **** *p* ≤ 0.0001).

Due to the lack of data on the CTX toxicity in the HEK293 cell line evaluated by the routinely used MTT method, firstly, it was necessary to evaluate the sensibility of this cell line to O/V. As shown in Figure 11A, after the cell exposure to O/V concentrations from 60/6 to 340/34 μM, a significant cytotoxic effect was detected from 140/14 μM O/V, highlighting that, as well as for other cell lines, O/V caused the cell damage at these concentrations. Therefore, the in vitro toxicity of the ciguatoxin was tested directly on this line, exposing the cells only to the CTX and measuring the cell viability by the MTT assay. As shown in Figure 11B, the 24 h cell exposure to increasing CTX3C concentrations (from 0.0001 nM to 0.01 nM) did not cause any significant cytotoxic effect. However, when the cells were exposed to CTX3C concentrations of 0.1 nM and higher, the cell viability decreased up to 40% at 10 nM CTX3C, revealing that the HEK293 Nav cell line allows for the detection of the presence of CTXs at concentrations from 0.1 nM without the need to use any other compound to exacerbate the CTX toxicity. With the aim to obtain a lower detection limit and increase the sensitivity of the CTX detection using the HEK293 cell line, the cells were exposed to a nontoxic O/V concentration for this cell line of 100/10 μM and increasing CTX concentrations. Figure 11C shows the cytotoxic effect of the cell coexposure to 100/10 μM O/V and increasing CTX3C concentrations. In this case, even at the lowest CTX3C concentration tested (0.0001 nM), a significant cytotoxic effect on the cells was detected, highlighting the fact that this cell line allows for the detection of the presence of ciguatoxins at concentrations as low as 0.0001 nM with O/V added at a concentration of 100/10 μM demonstrated as nontoxic.

The results obtained by electrophysiology and the MTT assay allows the HEK293 Na_v_1.6 cell line to be purposed as a potential alternative in the detection of CTXs since the detection limit is the lowest reported so far together with the 100/10 µM O/V that does not cause any cell damage.

A comparison between different cell-based assay techniques in terms of their limit of quantification (LOQ) is summarized in Table 1, illustrating that MTT in the HEK293 Na_v_ cells sensitized with O/V had the lowest LOQ.

## 3. Discussion

The search for a sensitive, reliable, fast and accessible method to detect the presence of ciguatoxins in samples has been a critical point over the years and has become more important in recent decades due to factors such as climate change which caused an expansion of the CTX-producing microalgae and the consequent expansion of CF to new regions [59]. Other factors such as the globalization of trade contributed to the increase in the cases of ciguatera poisoning intoxications in nonendemic regions [6]. Among all the methods used for CTX detection, the historically recognized and employed is the MTT assay on the N2a cell line [6]. However, this method has not been yet validated and established as an official reference method which makes it very difficult to compare the results obtained by different laboratories, since each uses its own assay conditions. Thus, many variations of this method using N2a as well as other different cell lines have been developed over the years with the aim to increase the sensitivity for CTX detection. In our work, the first limitation found when using the N2a cell line as a detection method for CTXs was their low sodium channel functionality as evidenced by the electrophysiological recordings and supported by previous data indicating the amplitude of sodium currents in this cell line ranging from −200 to −400 pA [33,34,35,60], underscoring the need for caution in the interpretation of cytotoxicity data derived from the use of neuroblastoma cells.

The lack of the cytotoxicity of CTX3C in the N2a cells treated with CTXs alone evaluated by the MTT method was previously reported [10,12,31,48], and the way to increase the cytotoxicity of ciguatoxins in this assay was the incubation of the cells at a nonstandardized concentration of O/V. The main problem is the cytotoxicity of the O/V treatments in the N2a cells which is also well documented [12,24]. Even so, different O/V concentrations as high as 500/50 µM O/V [30,61] were used when lower concentrations were proven to be highly toxic to cells [12]. Separate cell exposure to O and V also confirmed that V did not affect cell viability, a fact that could be explained since V acts on sodium channels, which are very scarce in neuroblastoma cells, being less toxic for the N2a cells than O, which acts on the Na^+^/K^+^ ATPase pump. In this study, an O/V concentration of 100/10 µM considered nontoxic was chosen. This O/V condition has been commonly used in recent assays with a generally accepted 20% reduction in cell viability to a dynamic range of 80% of cell viability [12,24,32,48]. Even though it has always been assumed that the N2a cells sensitized with O/V can detect the CTX effect on sodium channels assuming that the cytotoxicity observed by the MTT assay on N2a is due to the direct effect of ciguatoxins on the sodium channels; in this work, we demonstrated that the exposure of the N2a cells to 100/10 µM O/V for 24 h did not increase the sodium channel expression, not allowing for the detection of ciguatoxins due to their direct effect on the sodium channels, contrarily to what has been historically assumed and even patented [62]. Through the MTT assay under these conditions, the commonly used method was replicated obtaining similar cytotoxicity results for CTX3C as in previous studies [9,12,49] with a similar IC_50_. However, when the cytotoxicity under the same assay conditions was evaluated using different methods, such as the LDH and Alamar blue assays, that did not require washing or medium removal, the results obtained were significantly different, not allowing for the detection of the CTX cytotoxicity at any of the studied concentrations. The immunocytochemistry images showed alterations in the actin cytoskeleton in the treated cells, according to previously reported results for ouabain [55] and ciguatoxins [51]. When the cells were exposed to a combination of these two compounds that cause alterations in the actin cytoskeleton, the damage was much more pronounced.

These data, together with the microscopic observations of the cell weakening and detachment after the treatment and the confocal microscopy images that confirmed the damage in the actin cytoskeleton as well as the electrophysiological recordings showing a very low rate of functional sodium channel expression, allow us to propose that the cytotoxic effects of CTX3C obtained by the MTT assay are not really quantifying the effects of ciguatoxins on their main target, the sodium channels. The N2a cells, although still viable but with the cytoskeleton damage, are not well adhered to the wells; therefore, the processes of washing, removal of the medium and incubation with shaking that the MTT method requires [11,63] make these still viable cells detach from the bottom of the wells, resulting in a false decrease in cell viability. This hypothesis is supported by the fact that alternative cytotoxicity detection methods, such as the Alamar blue or LDH assay, did not detect any additional decrease in the cell viability higher than that caused by the concentration of 100/10 µM O/V.

Although the main effect of forskolin is the activation of adenylate cyclase, the interaction of forskolin with sodium channels was reported as eliciting an increase in the sodium current amplitude in different cell lines [37,39,40]. Moreover, this molecule was reported as a promoter of neuroblastoma cell differentiation [36,42]. Therefore, the 24 h N2a cell exposure to 10 µM forskolin led to an increase in the N2a sodium current amplitude resulting in an increase in the CTX cytotoxicity under the same assay conditions as in both alternative methods studied, the LHD and Alamar blue assays. Moreover, in the MTT assay, despite obtaining a similar IC_50_ due to following the same assay procedure, higher sensibility was obtained in the forskolin-pretreated cells represented by a decrease in the cell viability up to 81.2 ± 2.3% (*n* = 4) with 10 nM CTX3C and 100/10 µM O/V compared to the nonpretreated cells where the cell viability under the same conditions decreased only up to 66.1 ± 3.6% (*n* = 4). Therefore, the data presented in this work demonstrate that the N2a cell pretreatment with 10 µM forskolin for 24 h enhances the N2a cell sensitivity to CTXs. 

Since the N2a cell line is routinely used and recommended to monitor the presence of CTXs [6], all the results presented in this report led to the question whether this cell line was suitable for the detection and quantification of compounds whose main targets are the sodium channels, such as ciguatoxins. Since, as we demonstrate in this work, the direct effect of CTXs on the sodium channels is not being quantified, which has more serious consequences than the effects produced on the cytoskeleton, this leads to the under- or overestimation of the real CTX effect. Given the problems of the MTT assay on N2a, multiple alternative detection methods for voltage-gated sodium channel modulators have been investigated, involving diverse approaches, from the use of different cell lines [23] to the use of different assays based on the CTX ability to depolarize cell membrane [25] or receptor binding assays [28,64]. One of the alternatives proposed was the human neuroblastoma SH-SY5Y cell line [23], assuming that it would better mimic the effects caused by CTXs in the human nervous system. However, electrophysiological experiments corroborated the finding that the SH-SY5Y cell line, as well as N2a, did not show any functional sodium channels, which was in accordance with the previously reported results for the same cell line [56]. Despite being suggested as an alternative for CTX detection, the SH-SY5Y cell exposure to 10 nM O and 25 µM V decreased the cell viability by 25% compared to the control conditions, and the addition of 0.5 to 10 nM CTX3C only decreased the cell viability by 26 to 30% even at the highest CTX3C concentrations [23], indicating that this cell line allows neither for the detection of CTXs at low limits nor for their quantification. Similar results were obtained in our study where the decrease in the cell viability elicited by 100/10 µM O/V hardly increased even at the highest CTX3C concentrations, a fact that could be explained by the low sodium channel functionality in the undifferentiated SH-SY5Y cells. Therefore, the detection of ciguatoxins determined by the decrease in cell viability employing the O/V pretreatment may not be appropriate when using the human neuroblastoma SH-SY5Y cell line.

In contrast, in the HEK293 cells stably expressing the Na_v_1.6 alpha subunit, CTX3C alone, at concentrations of 0.1 nM and higher, decreased the cell viability, reaching a 40% cell death at 10 nM CTX3C. This fact reveals that the HEK293 Nav cell line allows for the detection of ciguatoxins at concentrations from 0.1 nM CTX3C due to their true effect on the sodium channels without the need to use any other compound to exacerbate the CTX toxicity. This is a considerably low limit since no additional compounds are used to detect cytotoxicity by the MTT assay either in the SH-SY5Y or N2a cell lines. Moreover, when the MTT assay was performed in this cell line replicating the N2a conditions, the detection limit decreased up to 0.0001 nM CTX3C in the presence of O/V at a concentration of 100/10 μM demonstrated as nontoxic [12,49]. This fact reveals that the HEK293 Nav cell line constitutes an alternative for detecting the presence of CTXs at considerably low concentrations either with or without O/V. 

## 4. Materials and Methods

### 4.1. Toxins and Drugs

Noncertified CTX3C was obtained from Wako (FUJIFILM Wako Germany) and dissolved at a concentration of 1 μM in DMSO. Serial toxin dilutions were performed in Locke’s buffer solution containing (in mM) 154 NaCl, 5.6 KCl, 1.3 CaCl_2_, 1 MgCl_2_, 10 HEPES and 5.6 glucose (pH 7.4). Forskolin was purchased from Sigma and dissolved in DMSO at a final concentration of 25 mM. Ouabain and veratridine were purchased from Thermofisher and dissolved in DMSO at a final concentration of 200 nM and 5 mM, respectively. MTT was purchased from Sigma, lactate dehydrogenase (LDH) and Alamar blue were both from Thermofisher.

### 4.2. Human and Mouse Cell Lines

Three different cell lines were employed and maintained at 37 °C in a humidified atmosphere with 5% CO_2_ and 95% air, replacing the medium every 2–3 days.

#### 4.2.1. N2a Mouse Neuroblastoma Cell Line 

N2a cells were obtained from the American Type Culture Collection (CCL-131) at passage numbers from 180 to 200 and a seeding density of 25 × 103 cells per 200 μL and maintained in RPMI-1640 medium supplemented with 1% glutamine, 10% foetal bovine serum and 1% penicillin/streptomycin for cytotoxicity testing. The cells were seeded in 96-well plates or on glass coverslips in 12-well plates for electrophysiology testing at a density of 70 × 103 cells/well.

#### 4.2.2. Human Neuroblastoma Cell Line SH-SY5Y 

Human neuroblastoma cell line SH-SY5Y was obtained from the American Type Culture Collection (ATCC CRL-2266), cultured in DMEM/F12 medium (Invitrogen) at a 1:1 ratio supplemented with 10% foetal bovine serum and 1% GlutaMAX, both from Gibco, and antibiotics (100 UI/mL penicillin and 100 µg/mL streptomycin). The cells were subcultured in 25 cm^2^ tissue culture flasks and seeded in 96-well microplates for experiments at a density of 12 × 10^3^ cells per 200 μL of culture medium for cytotoxicity testing or on glass coverslips in 12-well plates for electrophysiology testing at 70 × 10^3^ cells/mL.

#### 4.2.3. Human Embryonic Kidney Cell Line (HEK293) Cell Culture

HEK293 human cells stably expressing the human Na_v_1.6 alpha subunit of the sodium channels were kindly provided, under a material transfer agreement, by Dr. Andrew Powell (GlaxoSmithKline R&D, Stevenage, UK) at passage numbers from 33–34 to 40, cultured in DMEM/F12 medium supplemented with glutamax, MEM nonessential amino acid solution (Gibco, 1% *w*/*v*), 10% foetal bovine serum and 0.4 mg/mL geneticin (G418, Gibco) at a density of 30 × 10^3^ cells per 200 μL for cytotoxicity assays. For electrophysiology, cells were seeded at a density of 70 × 10^3^ cells/mL. Before testing, cells were kept at 30 °C for 24/48 h to improve the sodium channel expression [65].

### 4.3. Determination of Cell Viability

#### 4.3.1. MTT Assay 

MTT (3-(4,5-dimethylthiazol-2-yl)-2,5-diphenyltetrazolium bromide) test was used to determine cell viability after treatments at different CTX3C concentrations (0.0001 nM, 0.001 nM, 0.01 nM, 0.1 nM, 1 nM, 5 nM and 10 nM) added to the culture medium for 24 h. DMSO at 10% was used as death control. After treatment, cells were incubated with MTT at a final concentration of 500 µg/mL in Locke’s buffer containing 119 NaCl, 5.9 KCl, 1 CaCl_2_, 1.2 MgSO_4_, 1.2 NaH_2_PO_4_, 22.8 NaHCO_3_ and 0.1% glucose (pH was adjusted to 7.4 prior to use) for 50 min at 37 °C. After washing off excess MTT, cells were disaggregated with DMSO overnight, and cell viability was determined measuring the absorbance of the coloured formazan salt at 595 nm in a spectrophotometer plate reader. 

#### 4.3.2. Alamar Blue Assay

Cells were treated at the corresponding CTX3C concentrations, and at the end of the treatment, 10% *v*/*v* of alamarBlue^®^ was added. Cells were incubated with the toxins and alamarBlue^®^ for up to 48 h. The cytotoxicity was determined by fluorescence measurements using a FL600 microplate fluorescence reader (Bio-Tek Instruments, Inc., Winooski, VT, USA) set at 530 nm (excitation) and 590 nm (emission). Cell viability was expressed as % of the control fluorescence emission.

#### 4.3.3. Lactate Dehydrogenase Assay

CyQUANT™ LDH cytotoxicity assay kit was used to analyse cell cytotoxicity, following manufacturer’s instructions. Cells were cultured and treated for 24 h at the different CTX concentrations. After treatment, 50 μL of medium samples, by triplicate, was transferred to a 96-well flat-bottom plate, and LDH release was evaluated by reading the absorbance in a plate reader and subtracting the 680 nm absorbance value from the data obtained at 490 nm.

### 4.4. Immunocytochemistry

For immunocytochemistry control, 100/10 O/V- and CTX3C-treated N2a cells seeded on glass coverslips were washed three times with phosphate buffered saline (PBS, containing in mM 137 NaCl, 8.2 Na_2_HPO_4_, 1.5 KH_2_PO_4_ and 3.2 KCl) and fixed with 4% formaldehyde and 4% sucrose. Fixed cells were permeabilized with 0.1% triton X-100, and nonspecific fluorescence was blocked with 5% bovine serum albumin (BSA). To evaluate the 100/10 O/V and CTX3C effects on the cytoskeleton, cells were incubated with a monoclonal anti-actin mouse primary antibody (1:200, Millipore, Germany, diluted in PBS containing 2% BSA) overnight at 4 °C and washed three times with PBS. Afterward, cells were incubated with an anti-mouse fluorescent secondary antibody (Alexa Fluor 488, Thermofisher, at a 1:500 dilution) for 1 h at room temperature. Coverslips were washed three times with PBS and mounted in 50% glycerol/PBS, sealed with nail polish and analysed using a Nikon confocal laser scanning microscope (Nikon, Melville, NY, USA) with an ORCA-ER camera (Hamamatsu photonics, Shizuoka Prefecture, Japan).

### 4.5. Electrophysiology

Cells seeded on glass coverslips were placed in a recording chamber with 0.5 mL Locke’s buffer as an extracellular solution. Recording electrodes were fabricated with borosilicate glass microcapillaries (1.5 outer diameter, resistances from 5 to 10 MΩ) and filled with an intracellular pipette solution that contained (in mM) 140 CsF, 10 EGTA, 10 HEPES, 5 NaCl, 2 MgCl_2_, pH adjusted to 7.3. Cells were maintained at a holding potential (V_hold_) of −55 mV establishing the whole-cell configuration to ensure adequate equilibration between the internal pipet solution and the cell interior. Voltage-gated sodium currents were recorded at room temperature (20–24 °C) in a computer-controlled current and voltage clamp amplifier (Multiclamp 700B, Molecular Devices) and using the Digidata 1440A data acquisition system (from Axon Instruments, San Jose, CA, USA). Signals were sampled at 50 kHz after low-pass Bessel filtering at 10 kHz and analysed offline, using the pClamp 10 software (Axon Instruments). Compensation circuitry was used to reduce the series resistance error by at least 70%. The effect of CTXs on sodium channels was evaluated after 5 min exposure of the cells to the toxin. To record the activation of voltage-gated sodium currents (I_Na_), voltage steps from −80 to +80 mV (10 mV increments) were applied.

### 4.6. Statistical Analysis

All data are expressed as means ± SEM of n determinations. Statistical comparison was by Student’s *t*-test or ANOVA followed by Dunnet’s test; *p*-values < 0.05 were considered statistically significant.

## 5. Conclusions

The N2a mouse neuroblastoma cell line currently used for the detection and quantification of ciguatoxins in samples does not have functional voltage-gated sodium channels. Therefore, the MTT method cannot detect the direct effect of the ciguatoxins on the sodium channels as historically assumed. It is proposed that what is being detected is a decrease in cell viability as a consequence of the methodology used. Due to the actin cytoskeleton damage caused by CTXs and the O/V treatment, cells, although viable, detach from the well and are removed. It was demonstrated that the 24 h exposure of neuroblastoma to 10 µM forskolin increases the expression of the sodium channels in the N2a cells; thus, this method could be used as an alternative for detecting the CTX compounds. Furthermore, the HEK293 cell line transfected with the 1.6 alpha subunit of the sodium channels was capable of detecting the presence of ciguatoxins even without the OV treatment. Additionally, in the case of using O/V to sensitize cells, 100/10 µM O/V was demonstrated not to be toxic for the HEK293 cells and to allow for the detection of ciguatoxins at lower concentrations compared to the rest of the methods.

## Figures and Tables

**Table 1:** Limits of quantification of ciguatoxin CTX3C by the different cell-based assay techniques in ciguatoxin detection as a function of the cell line employed.

METHOD	LOQ (M)
MTT in N2a sensitized with O/V	1 × 10^−12^
LDH in N2a sensitized with O/V	No cytotoxic effect
Alamar blue in N2a sensitized with O/V	No cytotoxic effect
MTT in N2a sensitized with O/V and pretreated with 10 µM forskolin	1 × 10^−11^
LDH in N2a sensitized with O/V and pretreated with 10 µM forskolin	1 × 10^−12^
Alamar blue in N2a sensitized with O/V and pretreated with 10 µM forskolin	1 × 10^−12^
MTT in HEK293 Na_v_	1 × 10^−10^
MTT in HEK293 Na_v_ sensitized with O/V	1 × 10^−13^

## Data Availability

Data will be available upon request.

## Supplementary Materials

The following supporting information can be downloaded at: https://www.mdpi.com/article/10.3390/md21110590/s1, Figure S1: Cytotoxic effects of O and O/V combinations in N2a cells; Figure S2: Cytotoxic effects of O/V and increasing CTX3C concentrations in N2a cells cultured in the presence of 5% FBS and treated with the different compounds in the presence of 2% FBS; Figure S3: No cytotoxic effects of 24 h incubation with 10 µM forskolin evaluated by MTT, LDH and Alamar Blue; Figure S4: Current-voltage relationship of sodium currents in SH-SY5Y cells. Figure S5: Cytotoxic effects in SH-SY5Y cells of different O/V concentrations after 24 h treatment.
