# Supplementary material for: Mouse N2a Neuroblastoma Assay: Uncertainties and Comparison with Alternative Cell-Based Assays for Ciguatoxin Detection
Source: Mar Drugs. 2023 Nov 13;21(11):590. doi: 10.3390/md21110590 (PMC10672529; doi:10.3390/md21110590)
Supplement: Supplementary file 1 [file marinedrugs-21-00590-s001.zip › marinedrugs-2684477-supplementary.pdf]

Table of Contents

1. Figure S1……………..……………....................….…..…………………………….……….…....2

2. Figure S2……………..……………....................….…..…………………………….……….…....3

3. Figure S3……………..……………....................….…..…………………………….……….…....4

4. Figure S4……………..……………....................….…..…………………………….……….…....5

5. Figure S5……………..……………....................….…..…………………………….……….…....5

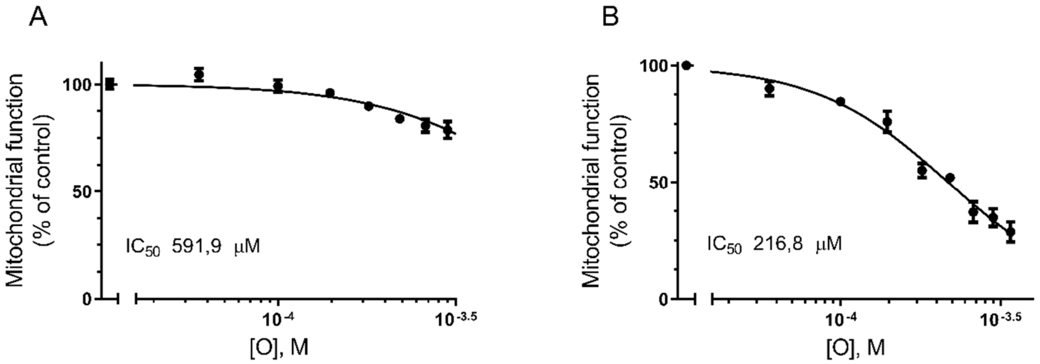

Figure S1: Cytotoxic effects of O and O/V combinations in N2a cells. A. Concentration response effect of 24 h exposure of cells to increasing O concentrations. B. Concentration response effect of 24 h exposure of cells to increasing O and V concentrations (60/6, 100/10, 140/14, 180/18, 220/22, 260/26, 300/30, 340/34 μM O/V).

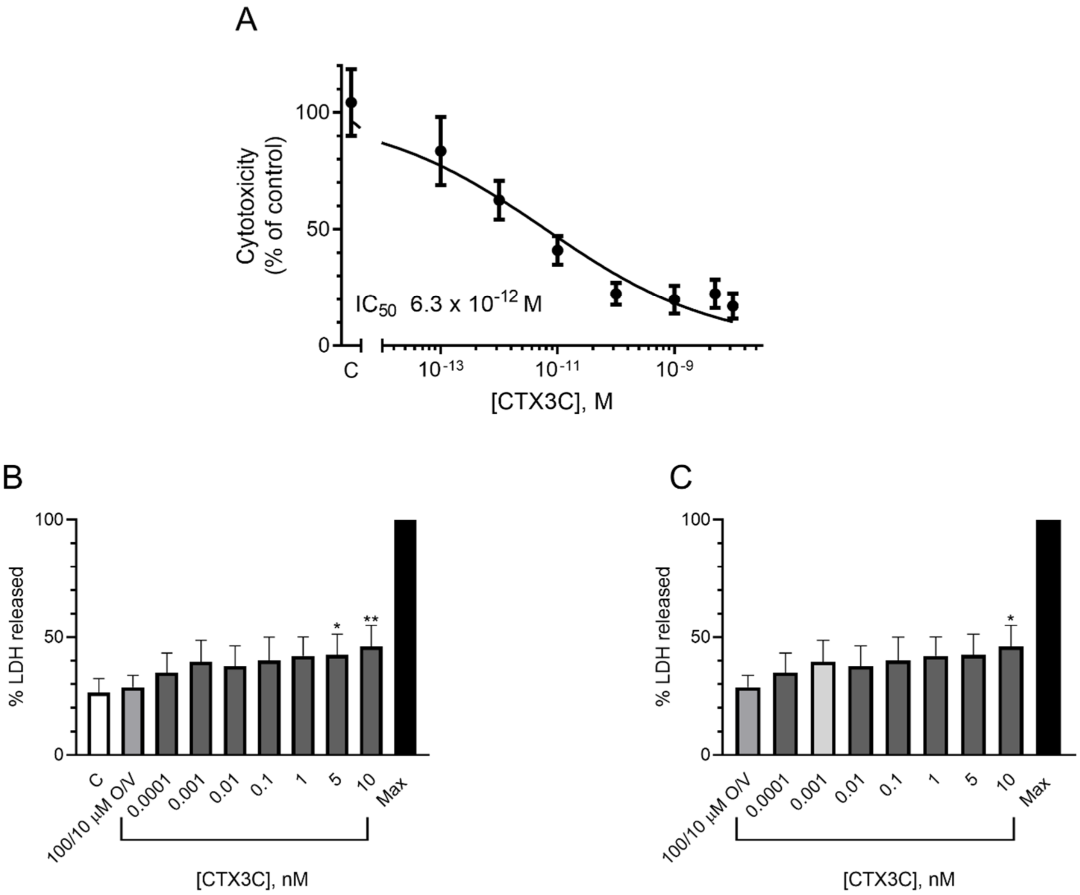

Figure S2: Cytotoxic effects of O/V and increasing CTX3C concentrations in N2a cells cultured in the presence of 5 % FBS and treated with the different compounds in the presence of 2 % FBSA. A. Concentration response effect of 24 h exposure of cells to increasing CTX3C concentrations and 100/10 μM O/V evaluated by the MTT assay. B. Absence of cytotoxic effects of O/V and increasing CTX3C concentrations in N2a cells evaluated by the LDH assay in contrast to control conditions. C. Absence of cytotoxic effects of O/V and increasing CTX3C concentrations in N2a cells evaluated by LDH assay in contrast to 100/10 μM O/V conditions. Cell viability data are expressed as percentage of untreated control cells, mean ± SEM of three independent experiments and cell. $^*p \leq 0.05$. $^{**}p \leq 0.01$.

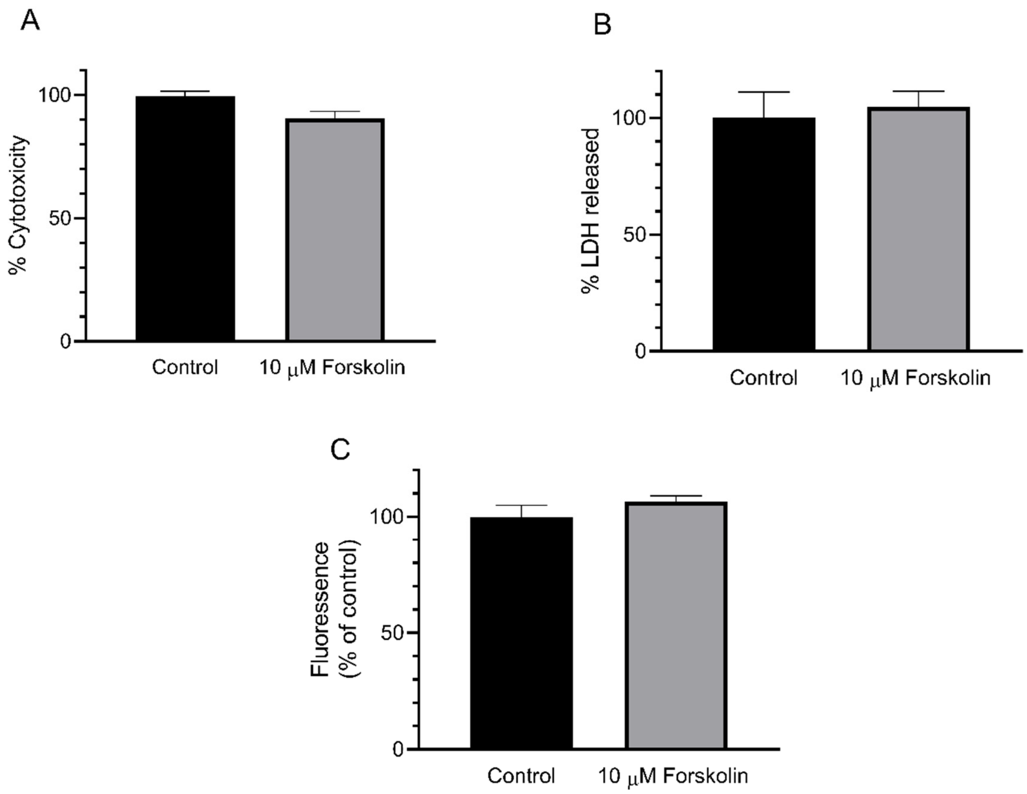

Figure S3: No cytotoxic effects of 24 h incubation with 10 μM forskolin evaluated by A. MTT assay. B. LDH assay. C. Alamar blue assay in the N2a cell line.

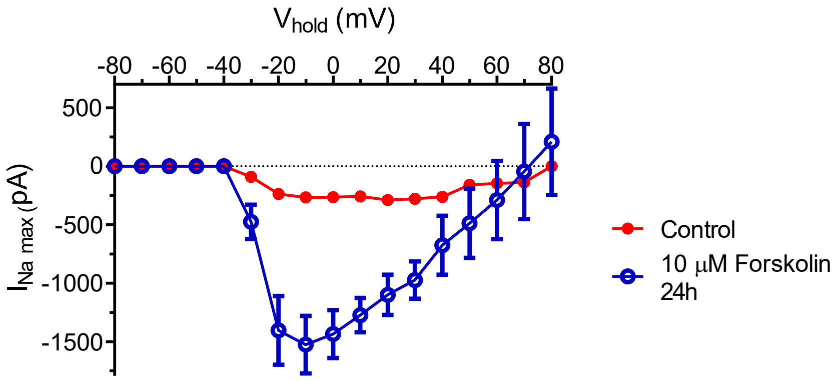

Figure S4: Current-voltage relationship of sodium currents in SH-SY5Y cells. Control cells (red) showed a maximum peak inward current of about –300 pA while cells seeded with 10 μM Forskolin and maintained 24 h showed an increase on maximum sodium current intensity up to -1500 pA.

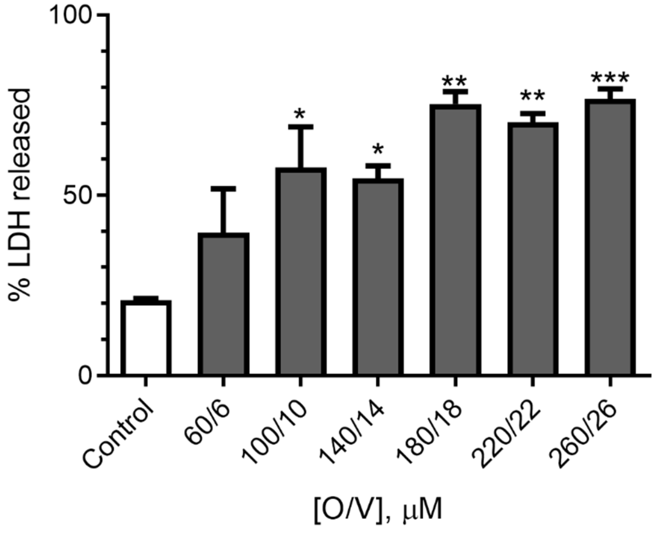

Figure S5: Cytotoxic effects in SH-SY5Y cells of different O/V concentrations after 24 h treatment.
